# Detecting Diagnostic Biomarkers of Alzheimer's Disease by Integrating Gene Expression Data in Six Brain Regions

**DOI:** 10.3389/fgene.2019.00157

**Published:** 2019-03-12

**Authors:** Lihua Wang, Zhi-Ping Liu

**Affiliations:** Department of Biomedical Engineering, School of Control Science and Engineering, Shandong University, Jinan, China

**Keywords:** Alzheimer's disease, biomarker discovery, gene expression, data integration, classification, machine learning

## Abstract

Alzheimer's disease (AD) is a neurodegenerative and progressive disease, which often causes irreversible damages to the cerebrum. The pathogenesis of AD is far from being fully understood, while there are some popular hypotheses. So far, the diagnosis of AD relies only on clinical screening in the form of imaging techniques or cerebrospinal fluid analysis, which may lead to inaccurate evaluation and then cause the delay of suitable treatments. While molecular biomarkers provide promising alternatives of establishing correct relationships between genotypes and phenotypes of clinical symptoms. In this paper, we propose a machine-learning-based method of identifying potential diagnostic biomarkers of AD based on gene coexpression network by integrating gene expression profiles in six brain regions. After building an integrated gene coexpression network of multiple brain regions, we decompose the differential network into some subnetwork modules. The module candidates from these coexpressed gene communities are then identified by screening their discriminative powers in control from disease samples. The potential biomarkers are then validated by multiple cross-validations and functional enrichment analyses. If the biomarkers successfully pass clinical significance tests, they can be used as a reference for clinical diagnosis after wet-experimental validations.

## 1. Introduction

Alzheimer's disease (AD) is a neurodegenerative and progressive disease, which causes irreversible damages to the cerebrum with cognitive and functional impairments (Porteri et al., 2017). Approximately, 50 million peoples are suffering from AD worldwide. The pathogenesis of AD is still poorly understood and some popular hypotheses have been proposed, such as genetics, cholinergic, amyloid and Tau protein hypothesis (Goedert and Spillantini, 2006). The progression of AD is rather long-time because its pathological change is a slowly accumulating process. It often takes years to decode, reveal and recognize the neuronal dysfunctions and neurodegeneration with dominant symptoms (Hardy and Selkoe, 2002; Goedert and Spillantini, 2006).

Currently, the diagnosis of AD generally relies on clinical screening in the form of imaging techniques or cerebrospinal fluid analysis (Jack et al., 2010). The limited dementia at an early stage often leads to inaccurate diagnosis and then results in the delay of beneficial treatments. Thus, the discovery of effective and efficacious biomarkers that can establish correct correspondences and relationships with clinical symptoms has become an urgent request (Porteri et al., 2017). Take it into consideration that the complicated genetic and environmental risk factors of developing AD in the human brain, there are thousands or 10,000 of candidates from genes, transcripts, and proteins with their interactions (Wang et al., 2016). It is a big challenge to identify AD biomarker molecules by making full use of the available big data. Due to the underlying complexity, network-based computational methods become important options to meet the challenge(Liu et al., 2011, 2012a).

In this paper, we aim to detect AD biomarkers by integrating gene expression data in six brain regions. Gene expression profiling data generates a genome-wide measurement of RNA abundance in parallel manners, which provide possible materials of bridging the gap between genotype and phenotype, which is the foundation of biomarker screening. Physiological and cellular processes are executed through interactions among genes and their products. Through the analysis of genetic network, which models their interactive activities, it is possible to screen out the core genes which play crucial roles in AD development and progression (Liu et al., 2009). Moreover, the incidence of AD in brain regions is sequential during disease progression. It is necessary to identify molecular biomarkers by integrating gene expression data from brain regions (Jack et al., 2013). To these ends, we provide a bioinformatics framework of detecting the potential diagnostic biomarkers based on differential gene coexpression network obtained by integrating gene expression profiles in multiple brain regions.

## 2. Methods

### 2.1. Framework of Biomarker Discovery

**Figure 1:**
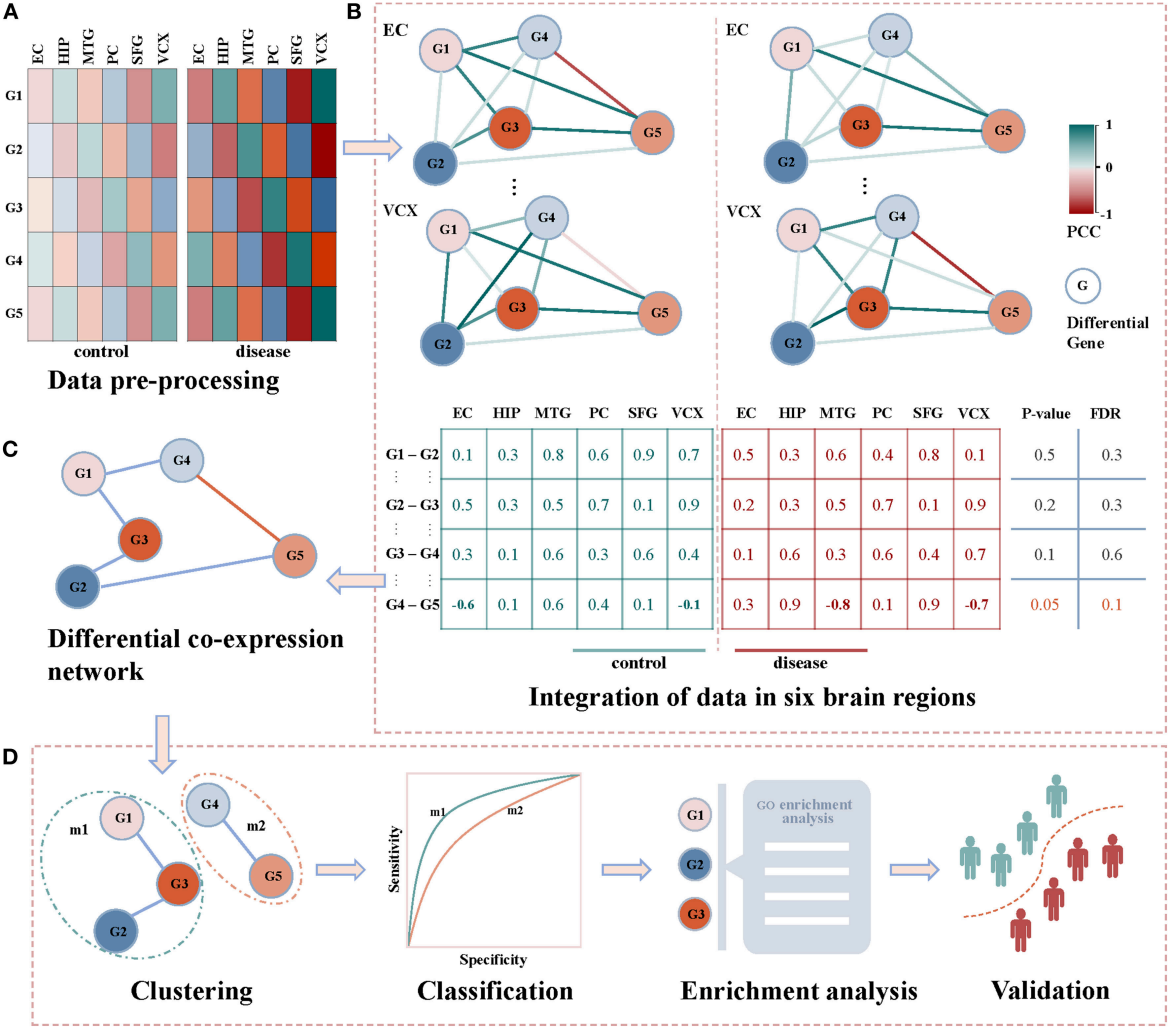
The framework of detecting AD biomarkers from gene expression data. **(A)** The gene expressions of AD samples and controls in the six brain regions of EC, HIP, MTG, PC, SFG and VCX respectively. **(B)** The union of differentially expression gene in each brain region. We generate the non-repetitive gene pairs from the pairwise differential genes. For each gene pair, we calculate their coexpression status in the control and disease samples via PCC. For the two correlation vectors, we implement Spearman's *t*-test to detect the differential gene coexpressions with thresholds of *p*-value ≤ 0.05 and FDR ≤ 0.1. **(C)** The differential correlation gene pairs construct a differential coexpression network. **(D)** The differential coexpression network is grouped into several subnetwork modules by clustering. They are screened out as candidate biomarkers when they successfully classify controls and diseases. The functional enrichment analysis will be performed to justify the dysfunctions underlying these candidates. Then, the validations in independent experimental settings are to check the classification performances of the identified biomarkers.

Figure 1 demonstrates our proposed framework of identifying diagnostic biomarkers of AD by integrating gene expression data in six brain regions. Briefly, we identify the correlation coefficients between differentially expressed genes across control and disease samples. By integrating the correlations of six brain regions, differential co-expressed gene pairs are selected by a statistical test, and they construct a differential co-expressed network. Then, we employ a network clustering method to partition off it into subnetwork modules. By evaluating their classification ability of distinguishing controls from diseases, the modules are screened individually by machine learning algorithms. The modules with the highest performance are identified as biomarkers after functional enrichment analysis and validation. The details shown in Figures 1A–D are introduced as follows.

### 2.2. Data Pre-processing

The microarray gene expression datasets are downloaded from NCBI GEO (ID:GSE5281) database (www.ncbi.nlm.nih.gov/geo) (Liang et al., 2007). The experiments contain the gene expression profiles of 161 samples in six brain regions, i.e., EC (entorhinal cortex), HIP (hippocampus), MTG (medial temporal gyrus), PC (posterior cingulate cortex), SFG (superior frontal gyrus), and VCX (primary visual cortex). In each brain region, there are the corresponding samples of disease and control simultaneously. The numbers of samples of affect/control cases are 10/13 in EC, 10/13 in HIP, 16/12 in MTG, 9/13 in PC, 23/11 in SFG, and 19/12 in VCX. According to the GPL570 annotation table, we map the probe set IDs to Entrez gene IDs and gene official symbols, respectively. When there are two or more corresponding gene IDs, we only select the one with maximum interquartile range. In each sample, the gene expression values are then normalized into *Z*-scores (Cheadle et al., 2003). Totally, there are 23,643 unique genes to get their expression measurements after data pre-processing.

### 2.3. Integration of Data in Six Brain Regions

#### 2.3.1. Differential Gene

First of all, we identify the differentially expressed genes in the six brain regions by the pre-processed gene expression data. Specifically, we evaluate the differential *p*-value of each gene across the control and disease samples via Welch's two sample *t*-test. For removing the high probability of committing type I error in multiple hypotheses testing, the corresponding FDR is also calculated (Noble, 2009). By setting up *p* ≤ 0.05 and FDR ≤ 0.01, we screen out these differential genes in each brain region respectively. We integrate the top 200 (top 10%) differential genes in each brain region and get the union of differentially expressed genes.

#### 2.3.2. Correlation Analysis

For building gene-gene coexpression relationships in multiple brain regions, we pick out the dysregulated interactions between genes using differential correlation analysis in each region individually. We firstly associate gene pairs in these identified differential genes in an all-against-all manner. In other words, we generate all the non-repetitive gene pairs that are produced by these differential genes. For each gene pair, we calculate their coexpression status in the samples via PCC (Pearson correlation coefficient) (Liu et al., 2012b), i.e.,

(1) $$\begin{array}{l} \text{r}\left(\text{X},\text{Y}\right)=\frac{\overset{\text{n}}{\underset{\text{i}=1}{\sum }}\left(X_{\text{i}}-\bar{X}\right)\left(Y_{\text{i}}-\bar{Y}\right)}{\left(\text{n}-1\right){\text{S}}_{X}{\text{S}}_{Y}}, \end{array}$$

where *X* and *Y* are the gene expression vectors. $\bar{X}$ and $\bar{Y}$ refer to the mean values of *X* and *Y*. *S*_*X*_ and *S*_*Y*_ represent their standard deviations. Then the coexpression values for all gene pairs in control and disease are obtained, respectively. We integrate the six coexpression values under control condition and those under disease condition into two new vectors across six brain regions. The differentially coexpressed gene pairs are identified via a nonparametric statistical testing. For the two vectors of six elements, we implement Spearman's *t*-test to detect the differential gene coexpressions with thresholds of *p*-value ≤ 0.05 and FDR ≤ 0.1.

### 2.4. Differential Co-expression Network

After collecting these differentially coexpressed gene pairs, we put them together to form into a differential coexpression network as shown in Figure 1C. It can be visualized when we import these dysregulated gene interactions into Cytoscape (Shannon et al., 2003). The subnetworks of this network will be targeted for identifying module biomarkers.

### 2.5. Clustering

For decomposing the whole differential coexpression network into subnetwork modules, we group the nodes by a network clustering algorithm, i.e., MCL (Markov clustering) (Van Dongen, 2000). Specifically, MCL algorithm is a fast and scalable unsupervised network clustering algorithm based on topological structures and features. It repeats two basic algebraic operations on matrices to simulate random walks on the network (Vlasblom and Wodak, 2009). The first operation is expansion, which is a process to calculate the probability of a random walk of length *n* between any two nodes in the network. Considering that the behavior of matrix multiplication is similar to random walks on graph, the Markov matrix associated with the graph can be used as the foundation of simulating these random walks. In a network, the flow is much easier within its dense regions than across its sparse boundaries. Thus, the second operation of MCL is inflation, which aims to keep this property by changing the distribution of each vertex transition values in the Markov matrix such that high values are further high and low values are further low. If the two-step iterations produce a convergent matrix, the final clustering will be achieved (Van Dongen, 2000).

### 2.6. Classification

These gene subnetwork modules provide the candidates for screening out the module biomarkers of classifying control and disease samples in brain regions. We perform an SVM (support vector machine) classification procedure to evaluate the discriminative ability of each module in distinguishing disease state from a normal state. SVM classifier aims to find an optimal hyperplane that satisfies the classification requirement and the optimal margin evaluation criteria are based on the distance between two support vectors (Suykens and Vandewalle, 1999). In the classification with two categories, the classifier can be constructed as follows. Given a training set of data points (*x*_*i*_, *y*_*i*_), i = 1, 2, ⋯ , *m*, ***x*** ∈ *R*^*n*^, *y* ∈ {±1}, optimal hyperplane H is:

(2) $$\begin{array}{l} \left(w\cdot x\right)+\text{b}=0. \end{array}$$

SVM classifier should meet some constraints, one of them is:

(3) $$\begin{array}{l} w\cdot x_{\text{i}}+\text{b}\geq 1,\;\text{if}\;{\text{y}}_{\text{i}}=+1;w\cdot x_{\text{i}}+\text{b}\leq -1,\;\text{if}\;{\text{y}}_{\text{i}}=-1 \end{array}$$

which is equivalent to

(4) $$\begin{array}{l} {\text{y}}_{i}\left[w\cdot x_{\text{i}}+\text{b}\right]\geq 1,\;\text{i}=1,2,\cdots \;,m \end{array}$$

The other is to maximize the margin which is calculated as 2/∥***w***∥. In other words, it is to minimize ***w***. For solving the constraint optimization problem, the Lagrange function is introduced:

(5) $$\begin{array}{l} L\left(w,\text{b},a\right)=\frac{1}{2}∥w∥-\lambda \left(y\left(\left(w\cdot x\right)+\text{b}\right)-1\right) \end{array}$$

Where λ_*i*_ > 0 is Lagrangian multiplier. By setting partial derivatives of (4) for ***w*** and *b* as 0, we finally find the optimal hyperplane and construct a classifier as:

(6) $$\begin{array}{l} \text{y}\left(\text{x}\right)=\text{sign}\left[\overset{\text{m}}{\underset{\text{i}=1}{\sum }}\lambda_{i}{\text{y}}_{i}{\text{x}}_{i}^{T}\text{x}+\text{b}\right] \end{array}$$

In the case of binary classification, we assess the classification performance of the SVM-based classifier by a leave-one-out cross validation (Cawley and Talbot, 2004). For a comparison study, we also implement several machine learning algorithms in the classification, such as naive Bayes, neural network and random forest (Liu, 2016).

### 2.7. Classification Evaluation

We evaluate the classification performance of these modules by the ROC (receiver operating characteristic) curves and their corresponding AUC (area under ROC curve) values. For each gene module, we compare the classification AUC values achieved by integrating gene expressions in six brain regions as well as in a single brain region. In addition, we also implement naive Bayes, neural network and random forest algorithms for classification. The comparison identifies the target module selected by SVM with the consistently high classification performance serving as AD module biomarkers. We also prove the rationality of data integration in six brain regions in the identification. The subnetwork module with highest AUC values is identified as the module biomarkers of AD for further cross-brain-region and cross-dataset validations. Then, the target network module with the best classification performances is regarded as the final identified AD biomarkers.

### 2.8. Enrichment Analysis

The functional implications of these network modules with good classification performance are obtained by GO (gene ontology) enrichment analysis. We implement our NOA (network ontology analysis) method (http://app.aporc.org/NOA/) to identify the enriched dysfunctions in these biomarker genes. From the functional implications, we can partially validate these identified biomarkers about their roles of AD development and progression.

## 3. Results

### 3.1. Differentially Expressed Genes

**Figure 2:**
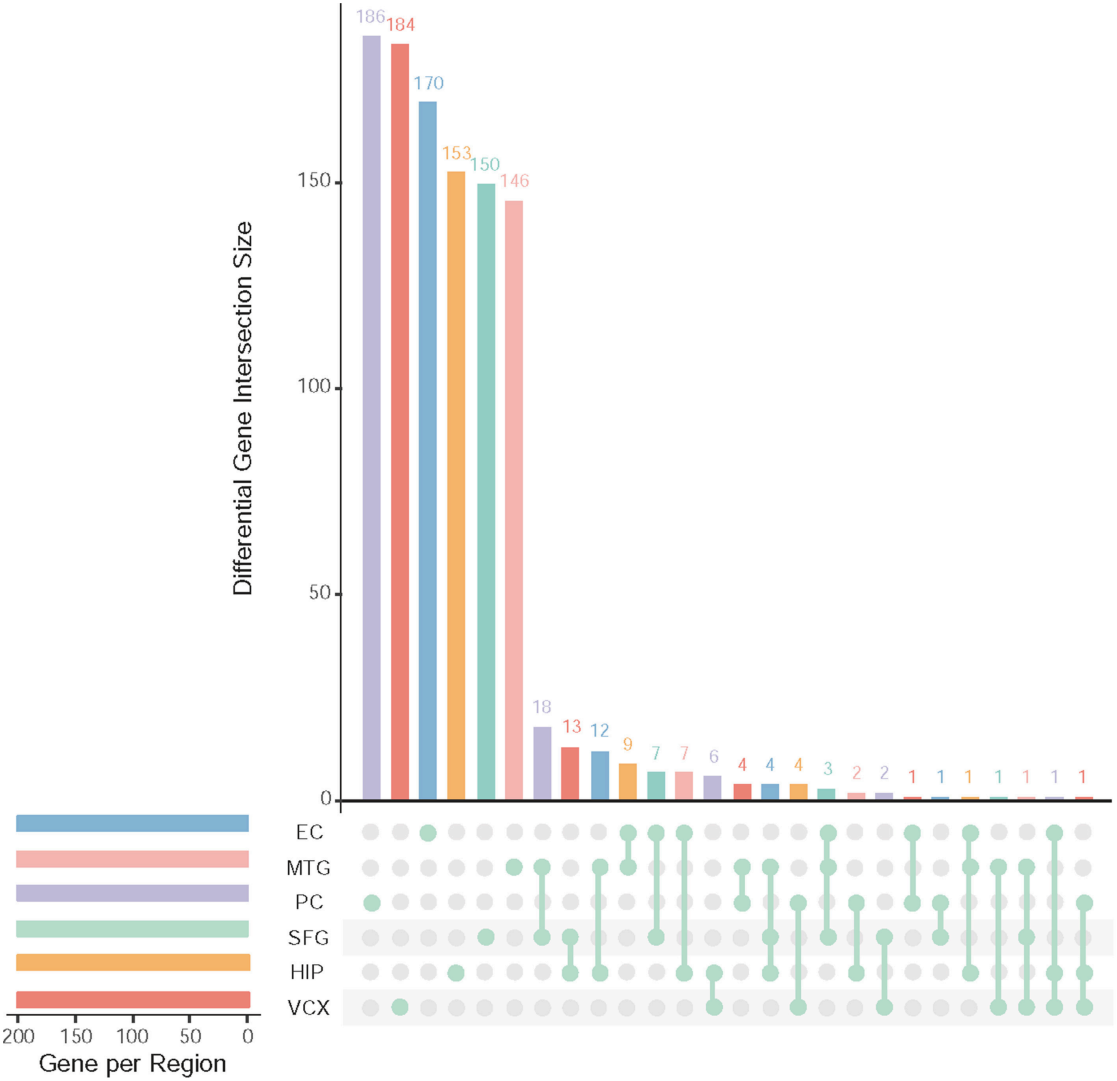
Top differentially expressed genes and their overlapping gene numbers in six brain regions.

After data pre-processing, we obtain 23,643 genes with their expression profiles. In each brain region, we identify the top 200 (top 10% genes picked after setting up *p* ≤ 0.05 and FDR ≤ 0.01) differential genes. All together, we identify 1,001 differentially expressed genes. Figure 2 illustrates the overlapping summary statistics of these differential genes distributed in the six brain regions. We find that most of the differential genes are only the differentially expressed genes in individual brain regions. Few genes are simultaneously differential across several brain regions.

### 3.2. Coexpression Network and Modules

**Figure 3:**
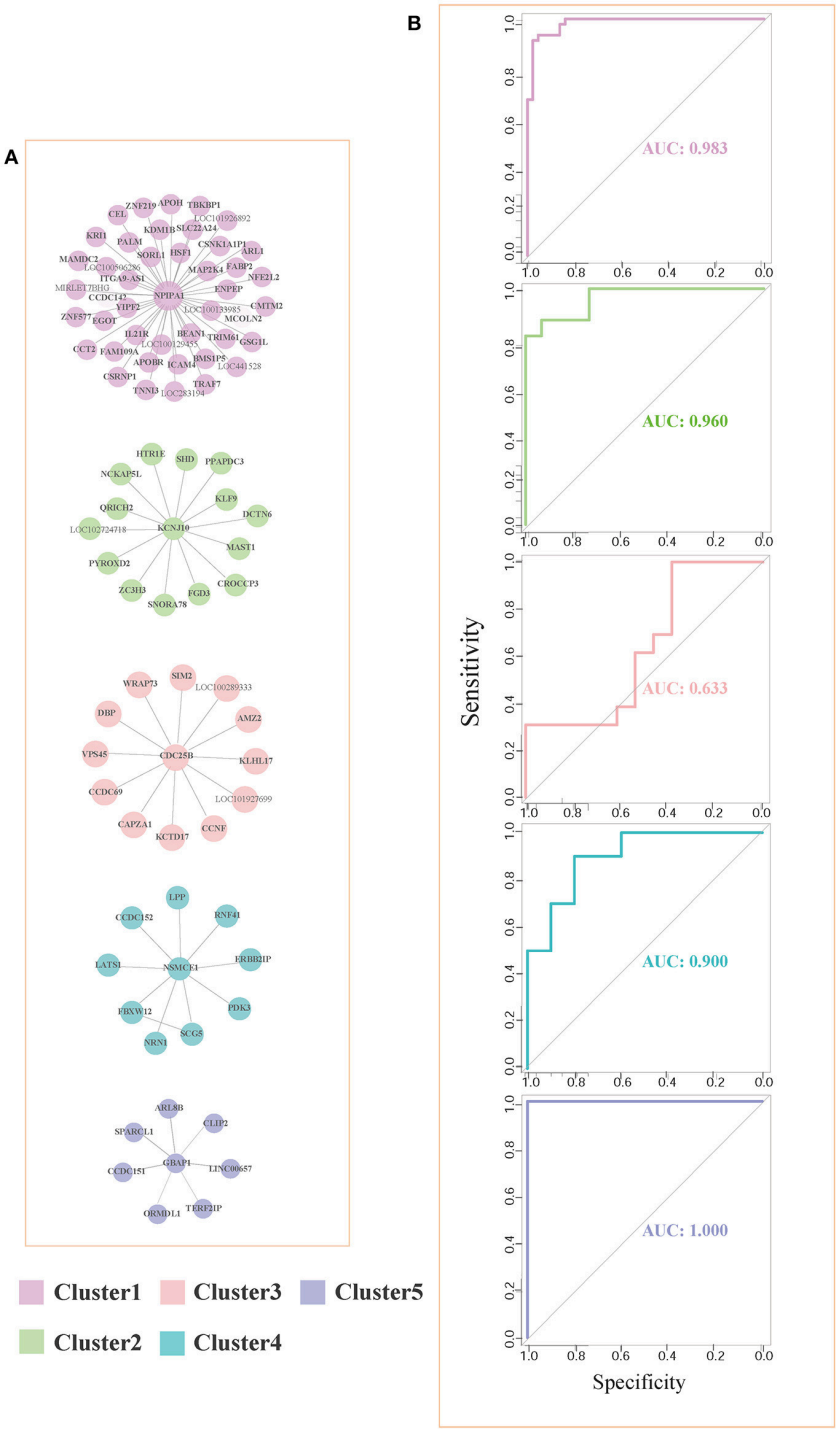
Five differential subnetwork modules and their ROC curves in classification. **(A)** Five gene modules identified by MCL clustering of differential coexpression network. Clusters 1–5 contain 44, 15, 13, 10, and 7 genes respectively. **(B)** The ROC curves of Clusters 1–5 in classifications in EC. The specificity and sensitivity are (0.955, 0.932), (0.933, 0.865), (0.385, 1.000), (0.800, 0.900), and (1.000, 1.000), respectively.

For each pair of differential genes, we calculate the differential correlation values via a statistical testing between control and disease samples. We identify the differentially coexpressed gene pairs and put them together to form a differential coexpressed network with 615 dysregulated interactions. By employing MCL algorithm, we identify some dysregulated subnetwork modules from the network. Figure 3A demonstrates five (top 5 number of genes in modules) of these modules. We note that there is obviously a hub gene in these modules individually, which indicates a topological feature of these differential coexpression networks.

As shown in Figure 3A, gene *NPIPA1* (nuclear pore complex interacting protein family member A1) is the identified hub differential gene with differential correlations with all the other genes in Cluster 1. *NPIPA1* is proved to perform biological functions of mRNA transport and protein transport. It has an interacting gene *MAP2K4*, which encodes an important membrane protein of MAPK (mitogen-activated protein kinase) family. From the interacting partners in Cluster 1, the biologically cooperative dysfunctions can be revealed. The differential coexpressed interaction between *NPIPA1* and *MAP2K4* implies the dysfunctional signal transduction in AD. From the network-based approach, the global scenario of dysfunctions is displayed for AD development and progression in the form of molecular subnetworks.

### 3.3. Biomarker Classification

For evaluating the performance of these clusters in distinguishing control and disease, we perform leave-one-out classifications. The ROC curves of these five clusters in the six brain regions are shown in Figure 3B. We also implement our evaluations in each brain region respectively. The sensitivity, specificity and AUC values are shown simultaneously. The detailed AUC values in six brain regions are shown in Table 1.

**Table 1:** The classification AUC values of the five clusters.

**Region**	**Cluster**				
	**Cluster 1**	**Cluster 2**	**Cluster 3**	**Cluster 4**	**Cluster 5**
ALL	0.983	0.960	0.633	0.900	1.000
Average	0.961	0.887	0.730	0.813	1.000
EC	0.938	0.938	0.331	0.890	1.000
HIP	0.953	0.956	1.000	0.730	1.000
MTG	1.000	0.609	0.988	0.550	1.000
PC	0.936	0.920	0.799	0.930	1.000
SFG	0.966	0.942	0.538	0.780	1.000
VCX	0.972	0.960	0.722	1.000	1.000

From Table 1, we find that the five clusters reach high AUC values in the six brain regions. The 5th cluster reaches the highest AUC values of 1.0. These results provide direct evidence for the effectiveness and efficiency of these candidate biomarkers in distinguishing between control and disease states. We also calculate the AUC values of summarizing these individual brain regions and their average values. The good classification performances indicate these modules can service as biomarkers of classifying the disease states in multiple brain regions. For better AUC values of these modules in various brain regions, we select Cluster 1 and Cluster 5 to further screening through different classification algorithms.

**Figure 4:**
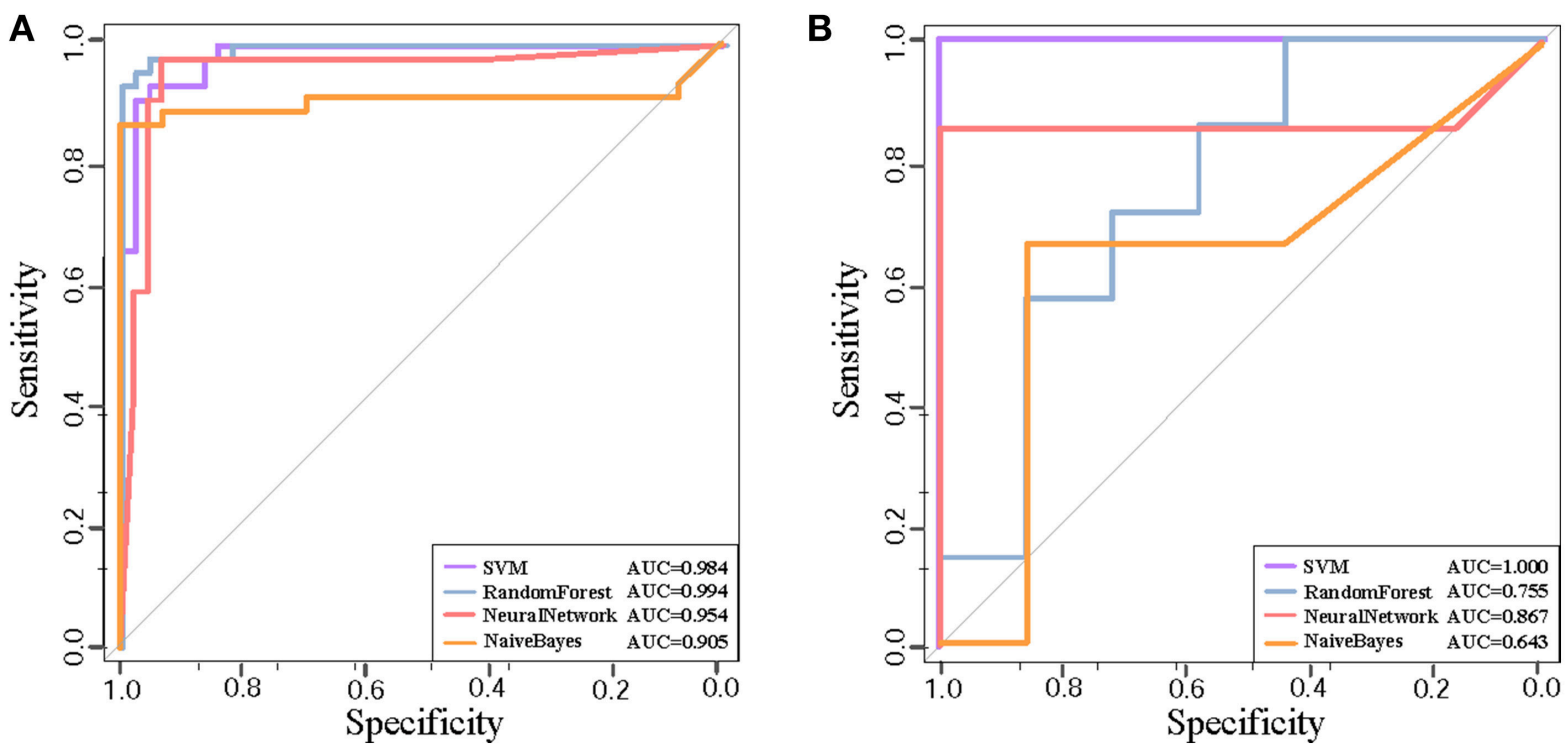
The ROC curves of four classification algorithms on Cluster 1 **(A)** and on Cluster 5 **(B)**.

We further test the discriminative capability of the two clusters by other three classification algorithms, i.e, naive Bayes, neural network, and random forest. Joint with SVM, Figures 4A,B demonstrate the ROC curves of the classifiers based on the four algorithms. In Cluster 1, we find that random forest achieves the best AUC of 0.994 from Figure 4A. While in Cluster 5, it achieves the AUC of 0.755 as shown in Figure 4B. Relatively, SVM obtains stably high AUC values of 0.984 and 1.0, respectively. Thus, we prefer SVM classifier to distinguish normal and disease states and Cluster 1 is the identified AD biomarkers.

**Figure 5:**
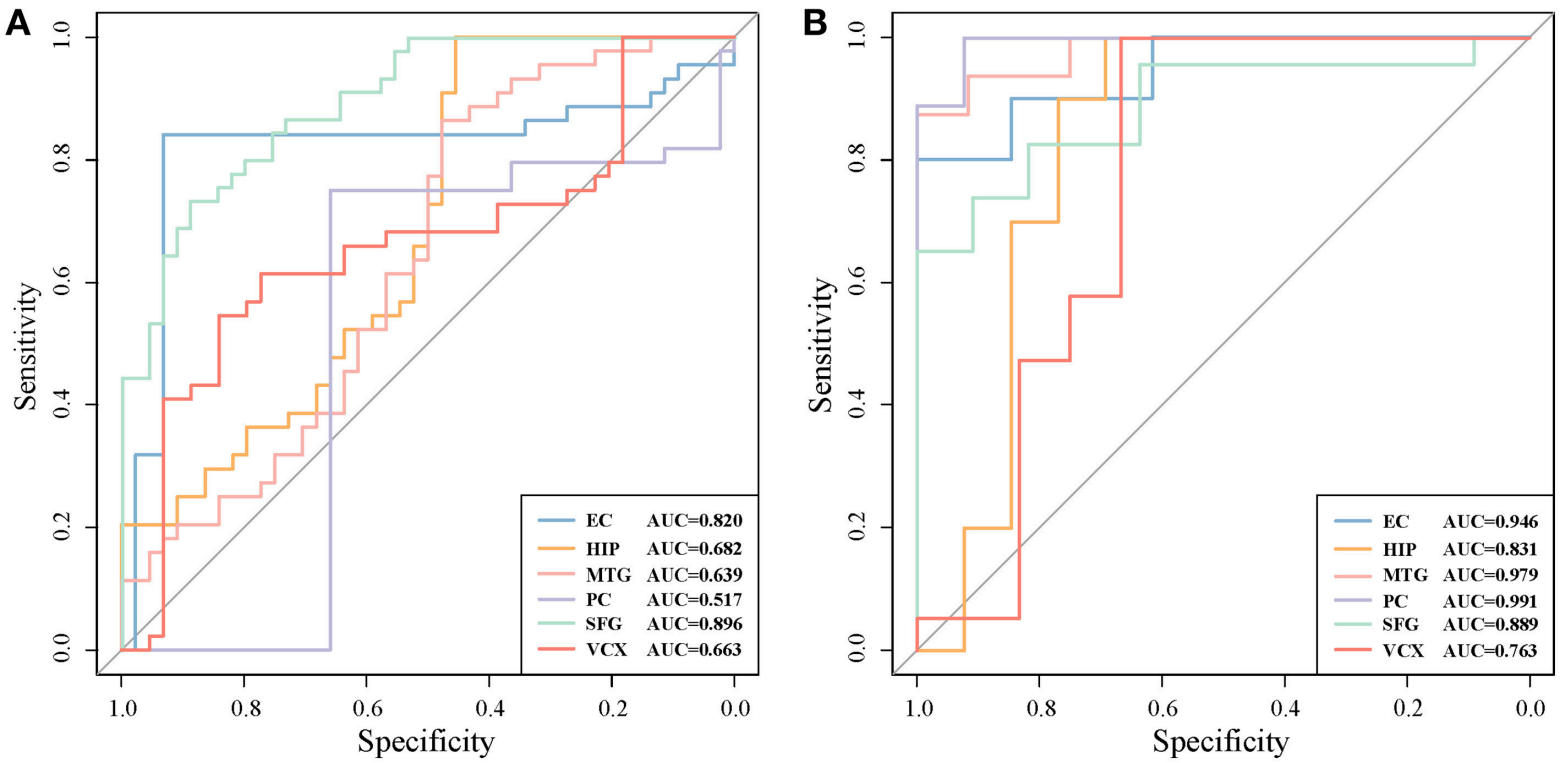
The classification performances by conventional biomarker discovery methods in the six brain regions. **(A)** The ROC curves of ‘DiffGene’ method on top 44 differential genes. **(B)** The ROC curves of ‘SVM-RFE’ method on top 1,000 differential genes.

For a comparison study with conventional biomarker discovery methods, we implement two widely-used methods, i.e., the method using differentially expressed genes (denoted as ‘DiffGene’ method) (Liu, 2016) and the variable/feature selection method by SVM-RFE algorithm (denoted as ‘SVM-RFE’ method) (Guyon et al., 2002). Figure 5 demonstrates the AUC values of classification results. As shown Figure 5A, the AUC values of ‘DiffGene’ method are not as good as our proposed method shown in Table 1. In Figure 5B, the AUC values of ‘SVM-RFE’ method are not consistently high. In brain regions of HIP, SFG and VCX, the AUC values of our proposed method (Table 1) exceed those of ‘SVM-RFE’. The comparisons demonstrate our method outperforms the conventional methods in terms of classification performance.

### 3.4. Biomarker Dysfunctional Analysis

For analyzing the functional implications in these identified diagnostic biomarkers of AD, we use NOA to enrich the GO annotations underlying these gene modules. Table 2 shows the significant GO terms of biological process. As shown in Table 2, we find the function of ‘lipid transport’ is enriched, which indicates the dysfunctional metabolism and energy transformation in AD. The epigenetics of ‘regulation of DNA methylation’ indicates the dysfunctional modifications related to AD. The important enrichments provide a functional map with blocks in these identified biomarker genes. They provide more evidence of functional importance of these biomarkers, which enlighten the insightful findings of AD pathogenesis.

**Table 2:** The enriched GO biological processes in the identified AD biomarkers.

**GO term**	**Representative gene**	**Term name**	**Corrected *P*-value**
GO:0006869	*CEL, FABP2, SORL1*	lipid transport	8.9E-5
GO:0050892	*CEL, FABP2*	intestinal absorption	2.6E-4
GO:0022600	*CEL, FABP2*	digestive system process	0.0015
GO:0018350	*CEL*	protein amino acid esterification	0.0016
GO:0044030	*TNNI3*	regulation of DNA methylation	0.0016
GO:0034196	*APOH*	acylglycerol transport	0.0016

## 4. Discussion

### 4.1. Cross-Region Biomarker Classification

AD is a chronic neurodegenerative disease which affects various brain regions of controlling various physical functions (Liang et al., 2007). The module biomarker of Cluster 1 with good classification power in control and disease samples has been identified by integrating gene expression data of six brain regions. It is of interest to investigate the cross-region classification performances for checking the potential pathogenic relationship between brain regions.

**Figure 6:**
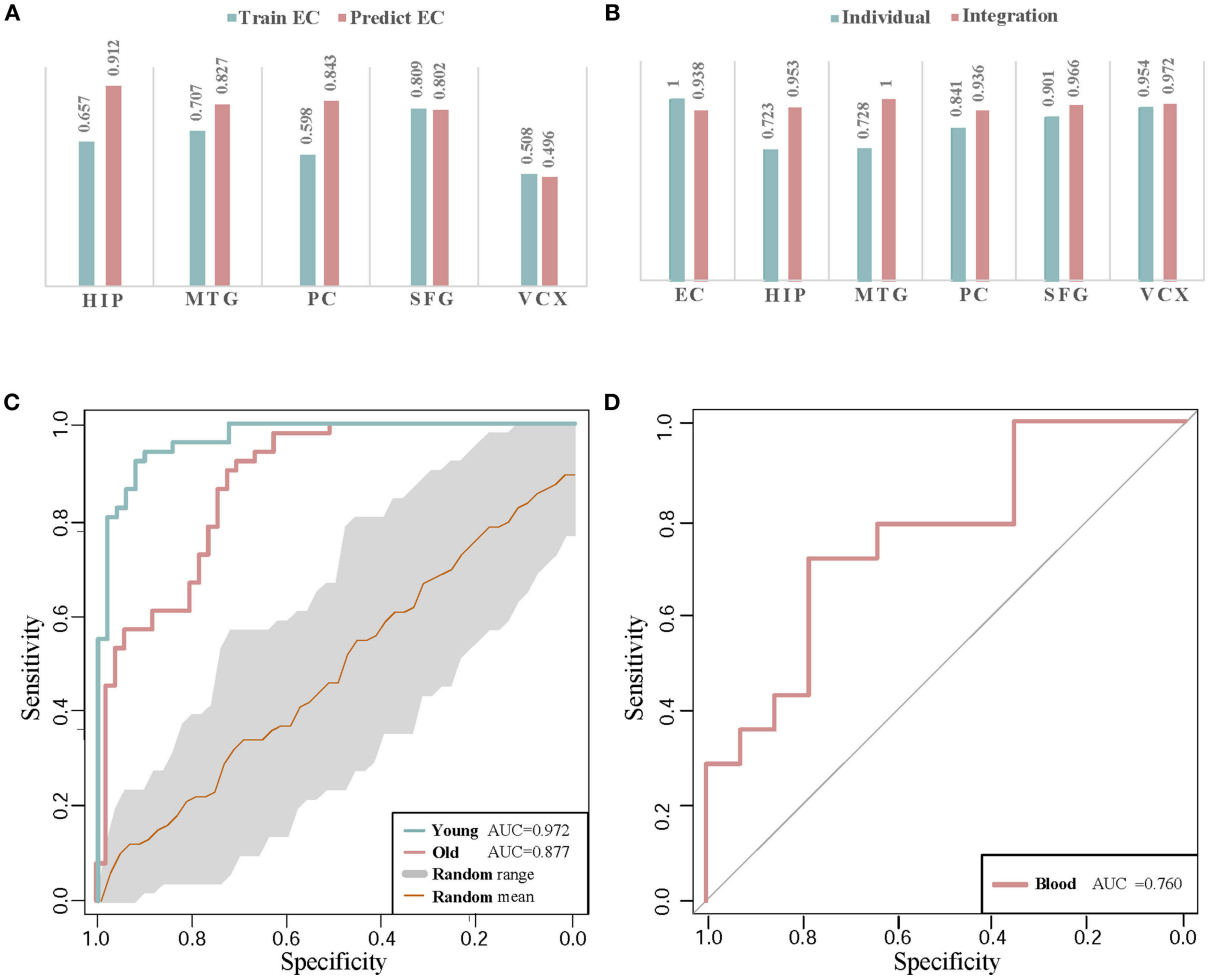
The classification validations of identified biomarkers. **(A)** The AUC values of cross-brain-region classifications between EC and the other five brain regions by module biomarker. ‘Predict EC’ means that we train the classifier by the gene expression of biomarkers in the other five brain regions respectively, and then test the classification performance in EC. And ‘Train EC’ means that we train the classifier in EC and test the classification in the other five brain regions respectively. **(B)** The classification AUC values obtained by individual-region and integration-region methods. The AUCs of the ‘individual’ module biomarkers are 1.000, 0.720, 0.719, 0.847, 0.902, and 0.954 in the six brain regions respectively. For the ‘integration’ module biomarker, the corresponding AUCs are 0.938, 0.953, 1.000, 0.936, 0.966, and 0.972, respectively. **(C)** The ROC curves of classification by AD biomarkers in independent datasets. ‘Young’ and ‘Old’ represent the datasets with different types of control sample respectively. The gray region refers to the standard deviations of classification in 30 random-choosing gene sets. **(D)** The ROC curve of biomarker classification in independent blood samples.

To evaluate the classification accuracy of module biomarker between six brain regions, we train the SVM classifier by utilizing gene expression data in one brain region and then test it in the other brain regions. Taking EC brain region as an example, we first extract the expression data of these module biomarker genes in EC and train the classifier for recognizing their patterns in control and disease samples. Then we test the trained classifier of distinguishing controls from diseases by the gene expression of these biomarker genes in the other five brain region individually. The five AUC values of classification are shown in Figure 6A. They are plotted as a bar. Secondly, we train the SVM classifier by the gene expressions in the other five brain regions, respectively and then test the classification performance in EC. The five AUC values are shown as the other bar graphs in Figure 6A.

From the AUC values of cross-brain-region validations, we can roughly estimate the dysfunctional relationships between the six brain regions from the view of dynamic gene expressions. In Figure 6A, we can find the classifiers achieve higher AUC values in HIP, MTG, PC, and SFG than that in VCX when we train them by the expressions of biomarkers in EC (0.657, 0.707, 0.598, and 0.809 vs. 0.508). This indicates the gene expressions in VCX are different from the other five brain regions. During AD progression in brain regions, the differences of effect in VCX have been identified (Liang et al., 2007; Liu et al., 2011). When we train the classifiers by the gene expression of biomarkers in the five brain regions, the classification performance for the samples in EC achieves high AUCs, i.e., 0.912 of HIP, 0.827 of MTG, 0.843 of PC, 0.802 of SFG, and 0.496 of VCX, respectively. We find the AUC of VCX is still the lowest one. This provides more evidence for the distinction of VCX during AD development. Moreover, the high AUC in some specific brain region implies its dysfunctional specificity. While we mainly focus on integrating the gene expression data of six brain regions to identify general biomarkers for AD instead of detecting specific biomarkers for individual brain regions.

Compared to the former AUCs by training the classifiers in EC and testing them in the other five regions, the higher AUC values prove the significant gene expression deviance of these biomarkers in EC. When we train the classifiers in the other five brain regions, the accurate classification performance in EC indicates that the gene expressions in the four brain regions contain the information of distinguishing controls from diseases. The asymmetric cross-brain-region classification results also inspire us to integrate the gene expressions in six brain regions to identify AD biomarkers for compensating the diversity of gene expressions in multiple brain regions.

### 4.2. Individual-Region Biomarker Classification

Instead of detecting AD biomarkers in the six individual brain regions, we integrate the differential coexpression gene pairs in these regions by a systematic strategy. For the comparison study, we also identify the candidate biomarkers by the gene expression data in the six brain regions individually and investigate their classification powers. We implement the whole former-described processes of biomarker discovery except the selection of differential gene coexpression pairs. In individual brain regions, the differential gene correlation pairs are alternatively based on the absolute difference values of the PCCs in control and disease samples. In each brain region, we rank the gene pairs according to differential correlations and select the same number of them as those in the former integration method. These differential gene pairs construct the individual gene coexpression networks in the six brain regions, respectively.

For each gene coexpression network, we also employ the MCL algorithm to decompose it to subnetwork clusters. For similarity, the clusters with the largest number of genes are recognized as the candidate biomarkers. For comparing the classifications of individual candidate biomarkers with the region-integrated biomarkers, we implement the leave-one-out cross-validations in these competitors and in the identified AD biomarkers.

Figure 6B demonstrates the comparison of AUC values in the six brain regions. By leveraging the gene expressions in each brain region, we implement the cross-validations of classification in the individual-region biomarkers and the region-integrated biomarkers. Except in EC, we can find the module biomarker achieves higher AUC values when compared to these candidate biomarkers in individual brain regions. In EC, the candidate biomarkers achieve a perfect AUC of 1.0 (vs. 0.938 of the identified biomarker). However, the identified module biomarker obtains higher classification AUC values than those in the other four individual brain regions. The results also indicate the rationality of identifying AD biomarkers by integrating gene expression datasets in several brain regions.

### 4.3. Cross-Dataset Biomarker Classification

For cross-dataset validation of our identified AD biomarkers, we also test their classification performance in independent datasets. The other AD gene expression profiles are downloaded from NCBI GEO (access ID: GSE48350). The dataset consists two sample-paired subsets in EC. One contains 15 AD brain samples and 21 control samples (from donors of young ages from 20 to 52). The other contains 15 AD brain samples and 18 control samples (from donors of old ages from 64 to 99). By utilizing the biomarkers, we test the classification in the two subsets, respectively. The ROC curves of classification by our module biomarker are shown in Figure 6C.

In classifying the AD samples with old-aged controls, the module biomarker achieves the AUC of 0.877. And the AUC value in the samples with young-aged controls is 0.972. The two AUC values prove the effectiveness and efficacy of our identified module biomarker in distinguishing AD samples from controls. Figure 6C also shows the ROC curve (with the gray range of standard deviations) in the same-size number of gene sets randomly choosing from the gene expression profiling data. The higher classification performances in the identified biomarkers provide more evidence for the efficiency and advantage of our proposed method.

### 4.4. Blood Validation

Currently, the accurate detection of AD in clinics is often based on nuclear magnetic resonance imaging, cerebrospinal fluid as well as PET (positron emission tomography) - CT (computed tomography). The finding diagnosis biomarkers provide possible alternatives with more clinical validations. Note that our identification is based on gene expression profiles in human brains. From a practical perspective in clinician, peripheral blood plasma testing is much more convenient, cheaper and with lower invasion in AD diagnosis (Suhre et al., 2017). Thus, we perform validation of these potential gene markers in blood gene expression samples to check their classification performances. The gene expression profiling data in blood mononuclear cells is downloaded from NCBI GEO (Access ID: GSE4226) (Maes et al., 2007). By mapping 44 genes in Cluster 1 to the measured blood gene expressions, we get 6 overlapping markers in blood samples of 14 AD patients and 14 normal controls. Using these six biomarker genes, the classification performance of ROC curve in distinguishing controls from diseases is demonstrated in Figure 6D. The AUC value achieves as high as 0.76. Although the number of biomarker genes measured in the samples is small, the diagnotic accuracy is competitive with the available clinic approaches. From the cross-dataset and blood validations, we partially verify the identified biomarkers in public data.

Recently, the circulating microRNAs in serum seem to be an alternative promising way of finding diagnostic biomarkers for complex diseases (Chen et al., 2017, 2018a). The development of computational methods for identifying potential diagnostic lncRNA biomarkers is also promising in the biomarker screening for AD, especially when these kind of high-throughput data are available (Chen et al., 2016, 2018b). It is an interesting research direction for AD biomarkers discovery from epigenetic transcripts in blood.

### 4.5. Relationship Between Biomarkers and AD Genes

**Figure 7:**
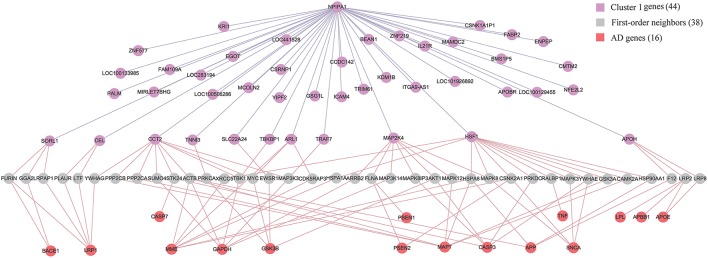
The relationship between biomarker genes in Cluster 1 and AD genes. After filtering the intersection of first-order neighbors of Cluster 1 and KEGG AD genes, we obtain 16 AD genes which have one-step indirect interaction with biomarker genes in Cluster 1.

Although *APP* (Jonsson et al., 2012), *APOE* (Morris et al., 2010) and *PSEN* (Hjermind, 2016) have been recognized as genetic risk factors of AD, we have not identified them in the diagnostic biomarkers because they are not differentially expressed genes in any of the six brain regions. It is of interest to study the relationship between biomarkers and AD genes. We firstly build up an integrative human protein-protein interaction (PPI) network by combining the interactions in various PPI databases (Liu et al., 2011). We employ the 28 genes in KEGG AD pathway as the documented AD genes (Liu et al., 2011). Then we identify the intersection of the first-order neighbors of the biomarker genes in Cluster 1 and those of AD genes. Figure 7 demonstrates their linkages. There are 16 AD genes containing the overlapping 38 first-order neighbors with the 44 biomarker genes. This indicates that the biomarkers have close relationships with these AD genes although they are not contained in the identified biomarkers. The results also prove the effects of AD causal genes have close distances with those biomarker genes in the molecular interactome.

## Conclusion

In this paper, we proposed a computational method of detecting AD biomarkers by integrating gene expression data in six brain regions. The framework is based on differential coexpression network and machine learning. The network modules are screened out by their classification powers via SVM classifiers. We identified five module candidates and regarded Cluster 1 as the identified AD biomarkers by using the other three classification algorithms for further screening. The cross-brain-region, cross-dataset, and validations in blood gene expression data provide evidence of its efficiency, efficacy, and advantage. Totally, 44 genes in Cluster 1 are targeted as the potential biomarkers in the form of a network module. Furthermore, the blood biomarkers are also important in clinical applications (Ngo et al., 2018), and we should screen out more genetic biomarkers from different datasets to map more potential blood biomarkers to improve classification accuracy. In the future, we also intend to incorporate these risky AD genes in our identification and investigate the causality between disease genes and marker genes. Considering the false positives in the computational strategy of identifying disease biomarkers, clinical validations of these potential biomarkers are urgent requests. If these identified AD biomarkers pass the multiple phases of clinical trials, they will be highly beneficial for early diagnosis of AD.

## Data Availability

The datasets analyzed for this study can be found in the NCBI GEO dataset: www.ncbi.nlm.nih.gov/geo/query/acc.cgi?acc=GSE5281.

## Author Contributions

Z-PL conceived and designed the study. LW wrote the code. LW and Z-PL analyzed the data and drafted the manuscript.

### Conflict of Interest Statement

The authors declare that the research was conducted in the absence of any commercial or financial relationships that could be construed as a potential conflict of interest.
